# Capturing the Features of Physical Activity in Old Adults during the COVID-19 Pandemic: Results of an Italian Survey

**DOI:** 10.3390/ijerph19116868

**Published:** 2022-06-03

**Authors:** Francesca Gallè, Elita Anna Sabella, Marco Di Muzio, Benedetta Barchielli, Giovanna Da Molin, Stefano Ferracuti, Giorgio Liguori, Giovanni Battista Orsi, Christian Napoli

**Affiliations:** 1 Department of Movement Sciences and Wellbeing, University of Naples “Parthenope”, Via Medina 40, 80133 Naples, Italy; giorgio.liguori@uniparthenope.it; 2 Inter-University Research Centre “Population, Environment and Health”, University of Bari Aldo Moro, Piazza Cesare Battisti 1, 70121 Bari, Italy; elita.sabella@uniba.it (E.A.S.); giovanna.damolin@uniba.it (G.D.M.); 3 Department of Clinical and Molecular Medicine, Sapienza University of Rome, Via di Grottarossa, 1015, 00189 Rome, Italy; marco.dimuzio@uniroma1.it; 4 Department of Dynamic, Clinical Psychology and Health, “Sapienza” University of Rome, Via degli Apuli 1, 00185 Rome, Italy; benedetta.barchielli@uniroma1.it; 5 Department of Human Neurosciences, “Sapienza” University of Rome, Piazzale Aldo Moro 5, 00185 Rome, Italy; stefano.ferracuti@uniroma1.it; 6 Department of Public Health and Infectious Diseases, “Sapienza” University of Rome, Piazzale Aldo Moro 5, 00185 Rome, Italy; giovanni.orsi@uniroma1.it; 7 Department of Medical Surgical Sciences and Translational Medicine, “Sapienza” University of Rome, Via di Grottarossa 1035/1039, 00189 Rome, Italy; christian.napoli@uniroma1.it

**Keywords:** physical activity, sedentary behavior, elderly, COVID-19

## Abstract

The restriction measures adopted to control the COVID-19 pandemic had significant consequences on individuals’ lifestyles. This study is aimed at assessing the amount and type of habitual physical activity (PA) in older adults during the advanced phase of the pandemic and their possible relationships with sociodemographic aspects. A questionnaire that included sociodemographic characteristics and the Physical Activity Scale for the Elderly (PASE) was administered online to elderly subjects living in the Apulia region, South Italy. A sample of 939 participants (57.1% F; mean age 75.9 ± 6.3) was obtained. In total, 68.8% of female respondents reported a decrease in PA during the pandemic, while 55.1% of men maintained their previous levels (<0.001). The total PASE score did not differ between gender groups (median value 91.7 in males vs. 90.0 in females; *p* = 0.067). However, differences were registered in leisure activities, particularly regarding walking (23.8 ± 14.8 in males vs. 20.2 ± 14.6 in females; *p* = 0.001). Higher PA levels were related with lower age (OR 0.253; 95% CI 0.192–0.333; *p* = 0.001). Since inactivity can affect elderly health and wellbeing, and considering the impact of the COVID-19 lockdown on this habit, health promotion strategies to counteract the negative effects of the pandemic should include interventions aimed at increasing PA in this sub-group of the population, especially among women and elderly subjects.

## 1. Introduction

Physical activity (PA) may contribute to maintaining and improving health and physical function. Conversely, physical inactivity is known as the fourth leading risk factor for global mortality and one of the leading risk factors for several non-communicable diseases and chronic conditions, such as cardiovascular, metabolic, and neoplastic diseases [1]. Physical inactivity is highly spread worldwide, and a significant part of the global population does not achieve the recommended amount of weekly PA for health [2]. In particular, in the elderly, PA can slow down the progression of diseases and improve the functions of daily living, increasing their autonomy and quality of life [3]; however, older adults usually show high rates of physical inactivity [2,4].

Since the beginning of COVID-19 emergency, the Italian Ministry of Health implemented specific control measures aimed to counteract the spread of infection, such as movement restriction, use of facial masks, and contact tracing, along with environmental control rules and routine microbiological checks [5,6,7]. The restriction of movement adopted to control the transmission of SARS-CoV-2 had inevitable consequences on individuals’ daily life [8,9,10]. Older adults were at higher risk of developing the severe form of the disease and experiencing its complications, with a consequent higher risk of death compared to other age groups [11,12]. Therefore, they were particularly invited to adopt restriction measures such as social distancing and self-isolation. Leaving work and being separated from family and friends had a significant impact on their behaviors and quality of life, as well as on their physical and mental health [13,14,15]. The partial or total interruption of habitual PA in old age can lead to a decline in functional and cardiorespiratory fitness and the deterioration of lipid and glycemic levels and psychological conditions, including in active subjects [16]. Reduced social and physical contact with others can lead to boredom, frustration, and a sense of isolation [17]. Moreover, prolonged stays at home may favor the adoption of sedentary behaviors, such as watching television, reading, and using mobile devices while sitting or lying down, which can lead to a decrease in self-reported physical and mental health and worse preexisting chronic conditions [18,19,20]. 

Aware of these issues, governments and institutions have promoted active lifestyles together with social distancing and self-isolation [21,22,23]. Even during the first phase of the pandemic, when a total lockdown was adopted as a control measure in several countries, different solutions for how to practice PA at home were proposed. In spite of this, many studies have reported a decrease in older adults’ PA during the SARS-CoV-2 pandemic [24,25], and some studies have reported that older adults had the lowest levels of PA among all age groups [14,26], and fewer changes in PA levels during the pandemic compared to other age groups [27], while in other they showed lower prevalence of insufficient PA during home-quarantine [28]. 

The PA level and its different components might be related to different social and individual aspects that should be known when PA promotion policies are planned, especially for elderly.

The present study is aimed at assessing PA levels and types in a sample of older adults living in the Apulia region throughout the advanced phases of the COVID-19 pandemic and highlighting possible relationships between the PA habitual behaviors and sociodemographic features of these individuals.

## 2. Materials and Methods

### 2.1. Setting and Participants

This cross-sectional study was performed during the period December 2021–February 2022 on ≥65-year-old participants living in southern Italy (Apulia region). The sample enrollment was performed by convenience, in accordance with the over-65-years population stratification in the 6 regional provinces [29]. People were invited to voluntarily participate in the survey by responding to a questionnaire. The target population of the study was composed of 909,396 residents over 65 years of age living in the Apulia region; thus, assuming a response proportion of 50% with a 95% confidence level, a minimum sample size of at least 384 individuals was estimated [30].

An Italian language questionnaire was used. The questionnaire was sent online to the participants to avoid paper usage and reduce the infective risk. The link to the online questionnaire was sent via social media or by email to the elderly subjects. Moreover, the link was sent to reference figures of recreational, religious, and cultural associations and facilities hosting self-sufficient elderly people (e.g., senior living communities that allow residents to do everything within the safety and comfort of a gated community) to allow a larger diffusion.

This study follows the World Medical Association Declaration of Helsinki principles, and it underwent to scientific and ethical evaluation by the Institutional Review Board of the Italian Inter-University Research Centre “Population, environment, and health” (final approval number 0530_2021).

### 2.2. Questionnaire

The questionnaire was divided into two sections. The first one was aimed at collecting sociodemographic information (e.g., age, gender), level of education (none/elementary/middle/high/university degree), and place of residence (community/institution). In order to calculate their body mass index (BMI), participants were also asked to report their values of weight and height. The weight status (underweight/normal/weight/overweight/obese) was evaluated according to the WHO international classification [31]. Respondents were also asked to self-report if their habitual PA increased, decreased, or remained the same during the pandemic compared to the pre-pandemic period.

The second section was represented by the Italian version of the Physical Activity Scale for the Elderly (PASE) in order to assess levels and features of participants’ PA. The PASE is a 12-item self-administered and validated questionnaire designed to investigate the duration, frequency, exertion level, and amount of physical activity in people 65 years and older (walking, recreational activities, exercise, housework, yard work, and caring for others) (Table 1) [32,33]. The PASE assigns a PA score (from 0 to 793, with higher scores indicating greater levels of PA) by evaluating the frequency, duration, and intensity level of activities performed in the previous week, and it is used for the PA national surveillance system in this group of population [34]. The frequency of participation in leisure activities, including walking outside home, light, moderate, and strenuous sport and recreation, and muscle strengthening, was categorized as never, seldom (1–2 days/week), sometimes (3–4 days/week), and often (5–7 days/week) performed. Duration of the activities was recorded as “less than 1 h”, “between 1 and 2 h”, “2 to 4 h”, or “more than 4 h”. Housework (light and heavy), lawn work/yard care, home repair, outdoor gardening, and caring for other people were recorded as “yes” or “no”. Paid or unpaid work that did not involve mostly sitting activities was reported as total hours per week. The individual total PASE score was calculated by multiplying the amount of time spent in each activity (hours/week) or participation (yes/no) in an activity by the item weights and summing over all the frequency products [32,33].

### 2.3. Statistical Analyses

A descriptive analysis was performed on the respondents’ sociodemographic, anthropometric, and behavioral characteristics. Age was expressed as mean values ± standard deviation (SD) and ranges. The number and percentage of respondents for the other individuals’ characteristics were reported. Comparisons between gender groups’ characteristics were performed using Student’s *t*-test for continuous variables and the chi-squared test for categorical variables. 

Due to their non-normal distribution, the median values of PASE total and partial (leisure time activities, household activities, and work-related activities) scores and interquartile ranges were calculated for the total sample and for gender and age groups. The differences in PA and its components between genders were assessed through the Mann–Whitney test. Male and female participants were also divided in age tertile groups to detect possible differences in their total PASE score through the Kruskal–Wallis test.

Spearman’s or Kendall’s correlation analyses were carried out to detect possible relationships between PASE score and quantitative (age, BMI) or qualitative (gender, educational level, place of residence) features of the sample, respectively. 

A logistic regression analysis was performed by considering the PASE total score (lower (0) or equal to/higher than (1) the median value) as the outcome, and age (dichotomized as lower or equal to /higher than the median value of 76 years), gender (males vs. females), educational level (lower than or equal to a degree), and place of residence (community or institution) as independent variables. The median values of the PASE score and age were chosen as measures of central tendency in order to have an equal distribution of the individuals in the lower half and in the higher half of the sample. Results were expressed as odds ratio (OR) values and 95% confidence interval (95% CI) values.

A *p*-value of 0.05 was assumed as a significance level. The software IBM SPSS version 27 for Windows (IBM Corp., Armonk, NY, USA) was used for data analyses.

## 3. Results

Overall, 939 questionnaires were completely filled in for this study and were included in the elaboration. Table 2 shows the main sociodemographic characteristics, weight status, and reported pandemic PA-related behavior of participants in the whole sample and grouped by gender.

The sample had a mean age of about 76 years and was mainly composed of highly educated, community-living, and overweight individuals. The male group included greater proportions of individuals with higher educational levels and higher BMIs than the female group. The largest part of the sample reported a decrease in habitual PA, although about 40% declared that they maintained their previous PA levels. In the gender comparison, a PA decrease was mainly reported by women. 

Table 3 shows the median total and partial PASE scores obtained from the whole sample and by gender groups.

A large component of habitual PA was represented by household activities for both male and female participants. Males showed higher engagement in leisure time activities than females.

**Figure 1:**
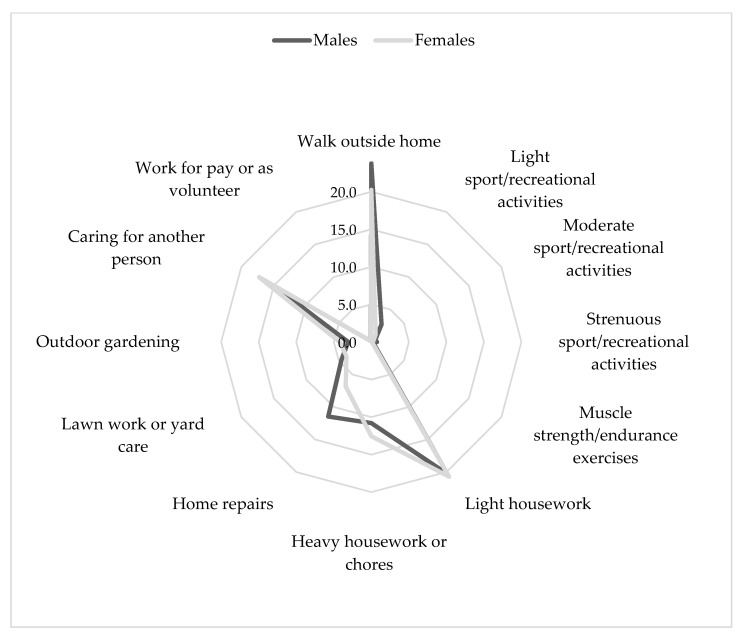
Types of physical activities reported by male and female participants.

The specific physical activity components reported by males and females are shown in Figure 1.

Although the two activity profiles show similarities regarding some domains, men were more engaged in home repairs and walking outdoors, while women seemed to be more involved in housework and as carers. Significant differences were found for walking (23.8 ± 14.8 in males vs. 20.2 ± 14.6 in females, *p* = 0.001), light sport/recreational activities (2.7 ± 6.9 vs. 1.2 ± 2.8, *p* < 0.001), moderate sport/recreational activities (0.6 ± 2.5 vs. 0.3 ± 1.5, *p* = 0.038), heavy housework or chores (10.8 ± 12.4 vs. 12.5 ± 12.5, *p* = 0.033), home repairs (11.5 ± 14.6 vs. 6.8 ± 12.6, *p* < 0.001), outdoor gardening (3.1 ± 7.2 vs. 4.2 ± 8.2, *p* = 0.027), and caring for another person (14.9 ± 17.3 vs. 17.2 ± 17.5, *p* = 0.046).

**Figure 2:**
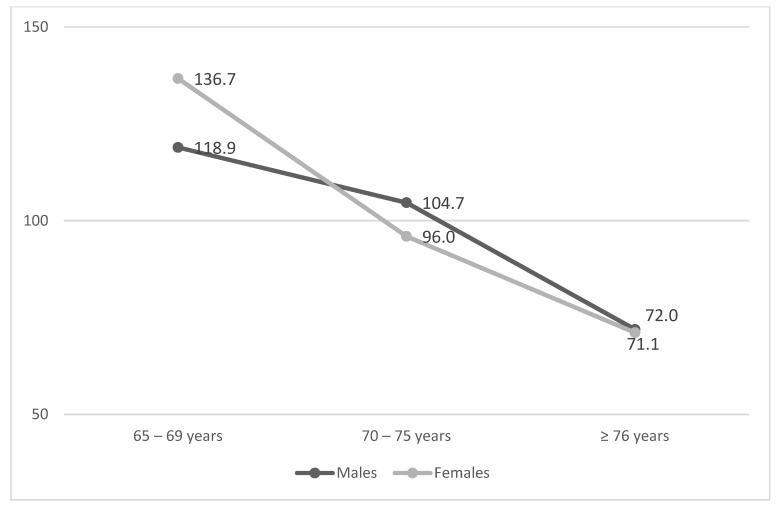
Total PASE scores by age group in male and female participants (*p* < 0.001).

Figure 2 shows the decrease by age group in the total PASE scores of male and female participants (*p* < 0.001 in both groups).

In the correlation analyses, only age (Spearman’s Rho −0.447, *p* < 0.001) and place of residence (Tau-b −0.314, *p* = 0.000) were found to be related to PASE scores among the variables considered. Neither gender (Kendall’s Tau-b −0.050, *p* = 0.067) nor educational level (Spearman’s Rho −0.037, *p* = 0.251), and BMI (Spearman’s Rho 0.052, *p* = 0.111) were found to be related with PA levels.

These findings were confirmed by the results of the logistic regression analysis shown in Table 4.

Even in this analysis, a higher PASE score was found to be related to lower age.

## 4. Discussion

Ensuring sufficient level of PA can play a crucial role in helping people to get a healthier lifestyle and better quality of life [25]. Especially in older adults, PA reduces the risk of developing multiple physical and health outcomes [34]. The pandemic has significantly impacted the health status, lifestyles, and levels of PA in older adults. As a matter of fact, in our study, the majority of the sample declared a decrease in their habitual PA. 

A worrying prevalence of overweight and obesity was found in about 80% of the sample, especially among males. These data are higher than those found in a previous study conducted on the same population in the pre-pandemic era (69.2%) [30] and also higher than those reported by the Italian surveillance system “Passi d’Argento” for older adults in the Apulia region during the period 2017–2020 (63.7%) [35]. This may represent a consequence of a worsening in behaviors related to energy balance in this age group during the pandemic. As a matter of fact, in the same period, “Passi d’Argento” also reported a decrease in the Italian elderly PASE score (94.2 to 93.5) [35].

In this context and also in our study, the PA level assessed by PASE (mean value 90.1) was lower than the minimum cut-off points (~140 for males and ~120 for females) that predict healthy waist circumference [36], and this suggests the need to encourage older adults from this population to be more active. Moreover, the evidence is not consistent with a previous investigation performed in 2019, before the pandemic, showing a satisfactory proportion of Apulian older adults meeting the WHO recommendations on PA for health [30], although, in that study, a different tool was employed to assess habitual PA.

With regard to the different components of PA, sports or recreational activities were less reported by our sample, while walking and light-intensity activities were the most common PA types for both genders. In particular, the mainly reported components were household activities for both males and females, which can be the consequence of the social isolation experienced by older adults during the pandemic. In fact, it has been demonstrated that older adults experience excessive worrying in emergencies such as the COVID-19 pandemic [25], and this may lead them to pursue a higher level of compliance with infection control measures, increasing their home confinements.

In addition, males reported significantly higher levels of walking, light and moderate sports/recreational activities, and home repairs than females. These findings are consistent with previous evidence reporting sex differences among older adults [37], including older adults living in the same geographical area as our survey [30]. In particular, walking outdoor seems to be the most maintained activity in males, accordingly to the previous results [30]. This aspect should be underlined, considering that walking, adopted as a coping strategy during the pandemic period, is also associated with psychological wellbeing [38]. As for the other types of PA, it is well known that vigorous-to-moderate PA levels may produce the greatest benefits in performing the activities of daily living and have the most significant health effects in older adults [39]. Therefore, attention must be paid to the poor levels of strenuous and moderate PA found in our sample, especially in females, in light of the specific consequences to their independence. Our finding is not consistent with those provided by Di Sebastiano et al., who reported in a sample of Canadian adults that moderate and vigorous PA (MVPA) returned to pre-pandemic levels only after 6 weeks of social distancing; no interactions were observed between age and PA over time for MVPA, while light physical activity (LPA) also remained significantly lower after 6 weeks of social distancing [14]. However, that study was conducted on a sample of non-older PA tracking app users, and this could have produced different findings.

In their study, Sjöberg et al. showed that the reduction of light-intensity activities found in their sample of Swedish older adults was related to psychiatric dysfunctions, while higher-intensity PA was related to poor social support [40]. Therefore, both psychological and social aspects should be considered as possible determinants of PA in this age group, especially during global emergencies such as a pandemic.

Generally, in the pre-pandemic era, a higher educational level was associated with greater adherence to regular PA [41]; this was not confirmed in our study, where the association was not significant. Instead, both the correlation and regression analyses confirmed the association between older age and higher inactivity in both genders, as reported in other studies related to the COVID-19 pandemic [28]. With regard to this, it should be noted that our age-group data mirror data coming from the Italian surveillance system for the years 2017–2020, which highlight a progressive reduction of PA with age (PASE score decreasing from 101.1 in individuals aged 65–74 years to 85.6 in individuals aged 75–84 years and older and to 66.4 in the >84 years group) [35]. This underlines the need to pay particular attention to elderly inactivity in the current situation.

Furthermore, in our study, higher levels of PA were found to be related to living in a community. However, the number of institutionalized individuals who participated in the survey was scarce and may have limited the strength of this finding. The literature shows that living in institutions may be associated with lower engagement in PA and occupational activities and with lower quality of life [42,43]. Both environmental and individual factors, such as fear of falling or past sedentary lifestyles, may represent perceived barriers to regular PA in this setting [44]. Since only autonomous institutionalized older adults were included in our study, the specific needs of this population group should be investigated to create a supportive environment and to plan effective individual interventions aimed at increasing their PA. 

Our data are more worrying considering that the present study was performed at an advanced stage of the pandemic; several information campaigns were launched after the first months of the pandemic, aimed at promoting healthy behaviors during the restrictions [45]. However, many factors may affect adherence to the advice, including communication means and instruments [46]. 

It should be noted that the existing literature examining, in depth, PA during COVID-19 in older adults is limited, and the available articles are more targeted at analyzing the mental and health effects of PA rather than evaluating PA types and changes over time in this population group. Instead, the role and type of PA are fundamental for human wellbeing, especially in the elderly. In fact, although there is no direct evidence that PA can prevent or support the treatment of COVID-19, promoting an active lifestyle is a key intervention to counteract unhealthy conditions such as immune dysregulation, metabolic pathways impairment, physical dysfunction, and mental distress, which may favor or precipitate disease [15].

The authors are aware of the limitations of this study. First, participants were enrolled by convenience sampling from one region of south Italy; thus, the generalizability of our findings may be limited. Secondly, the cross-sectional design of the study limits its validity in assessing temporal relationships between exposure and outcome. Since the survey was carried out only once, it was not possible to directly evaluate variations in PA-related behaviors across the pandemic; however, our results were only compared to previously published data referring to the same population. In addition, the method of administering the questionnaire may have hindered the participation of people with limited technological skills, representing a selection bias. Moreover, it should be considered that the anthropometric information was self-reported and could be inaccurately measured. At the same time, the levels of participants’ PA were measured through a questionnaire and not using objective instruments such as accelerometers; questionnaire-based and accelerometer-based physical activity measures correlate to psychosocial constructs differently [47]. Thus, accelerometer-based PA measurements should be preferred as they are a more objective way to capture the levels of physical activity. Finally, other variables linked to PA levels (e.g., level of knowledge concerning the disease, psychological or social factors) were not investigated in order to avoid an excessively lengthy questionnaire. This could have hidden important information.

In conclusion, few studies are reported in the scientific literature concerning PA in large samples of older adults in the advanced phase of the pandemic. Therefore, our results may be of practical significance for the implementation or improvement of health programs for the older population.

## 5. Conclusions

Since inactivity can have important consequences on people’s health and wellbeing, the invitation to be as active as possible, targeting specific subgroups of the population and focusing on specific issues, is a fundamental duty of health promotion. Promoting an active lifestyle in the course of the COVID-19 pandemic is a key intervention to counteracting both the social and health effects of isolation and inactivity, particularly among older adults. It is well known that the COVID-19 lockdown had a great effect on PA levels, and the interruption of the complete lockdown has not compensated for the reduction in PA in the elderly. Targeting interventions of PA promotion to older adults, especially to women and the elderly—such as simply enhancing indoor exercise or walking habits around the house—may help to reduce inactivity and the related high levels of BMI. In this way, promoting technology literacy, also in the elderly [25], can allow access to apps providing PA pathways. Moreover, at this stage, further promising initiatives, such as moving medicine, should be considered for their inclusion in routine clinical practice [34] in order to enhance active aging. To gain this challenging target, it is, therefore, necessary that both health policy and clinical practice support this population group in achieving the recommended levels of PA.

## Figures and Tables

**Table 1:** Items of the Physical Activity Scale for the Elderly (PASE).

Item	Days of Activity	Hours per Day of Activity	Answer
*Leisure time activity*			
Walk outside home	never	less than 1 h	
Light sport/recreational activities	seldom	1–2 h	
Moderate sport/recreational activities	sometimes	2–4 h	
Strenuous sport/recreational activities	often	more than 4 h	
Muscle strength/endurance exercises			
*Household activity*			yes/no
Light housework			
Heavy housework or chores			
Home repairs			
Lawn work or yard care			
Outdoor gardening			
Caring for another person			
*Work-related activity*			yes/no
Work for pay or as a volunteer		number of hours per week	

**Table 2:** Sociodemographic and anthropometric characteristics of participants.

Variable	Whole Sample *n* = 939	Males *n* = 403	Females *n* = 536	*p*-Value
age (mean ± SD, range)	75.9 ± 6.3 (65–95)	74.8 ± 5.7 (65–93)	76.7 ± 6.5 (65–95)	<0.001
educational level (*n*, %)				
elementary	28 (3)	9 (2.2)	19 (3.5)	0.016
middle	283 (30.1)	103 (25.6)	180 (33.6)	
high school	457 (48.7)	206 (51.1)	251 (46.8)	
degree	171 (18.2)	85 (21.1)	86 (16)	
place of residence (*n*, %)				
community	871 (92.8)	381 (94.5)	490 (91.4)	0.068
institution	68 (7.2)	22 (5.5)	46 (8.6)	
BMI (*n*, %)				
underweight	3 (0.3)	0 (0)	3 (0.6)	<0.001
normal weight	214 (22.8)	58 (14.4)	156 (29.1)	
overweight	467 (49.7)	222 (55.1)	245 (45.7)	
obese	255 (27.2)	123 (30.5)	132 (24.6)	
PA during pandemic (*n*, %)				
equal than before	380 (40.5)	222 (55.1)	158 (29.5)	<0.001
decreased	527 (56.1)	158 (39.2)	369 (68.8)	
increased	32 (3.4)	23 (5.7)	9 (1.7)	

BMI: body mass index; PA: physical activity.

**Table 3:** Frequency of weekly activities reported by participants.

Items	PASE Score Median Value (IQR)			
	Whole Sample *n* = 939	Males *n* = 403	Females *n* = 536	*p*
Leisure time activities	30.0 (25.0)	30.0 (30.3)	30.0 (27.8)	<0.001
Household activities	60.0 (30.0)	60.0 (30.0)	60.0 (35.0)	0.748
Work-related activities	0.0 (0.0)	0.0 (0.0)	0.0 (0.0)	0.775
Total PASE score	91.7 (50.0)	91.7 (35.0)	90.0 (52.8)	0.067

PASE: Physical Activity Score for the Elderly.

**Table 4:** Logistic regression model built on PASE scores as outcomes.

Variable	Odds Ratio (95% CI)	*p*
gender		
males	reference	0.224
females	0.839 (0.633–1.113)	
age		
<76 years	reference	<0.001
≥76 years	0.253 (0.192–0.333)	
educational level		
elementary to high	reference	0.137
degree	1.316 (0.916–1.889)	
place of residence		
community	reference	<0.001
institution	0.021 (0.003–0.155)	
BMI		
under/normal weight	reference	0.633
overweight/obese	1.083 (0.780–1.505)	

## Data Availability

All data presented are available upon request from the corresponding author (F.G.).
